# BvrR From *Brucella abortus* Induces Neuroinflammation Through IRE1‐Mediated Activation of ATF2 and NF‐κB

**DOI:** 10.1002/mbo3.70219

**Published:** 2026-01-21

**Authors:** Zhao Wang, Xinwen Yu, Boyu Liu, Dongni Ren

**Affiliations:** ^1^ Department of Experimental Surgery The Second Affiliated Hospital of Air Force Medical University Xi'an China; ^2^ Department of Emergency Medicine The General Hospital of Western Theater Command Chengdu China

**Keywords:** *Brucella abortus* BvrR, IRE1/ATF2, IRE1/NF‐κB p65, microglia, neurobrucellosis, neuroinflammation

## Abstract

*Brucella*‐induced neuroinflammation represents a key mechanism in the development of neurobrucellosis. The objective of this investigation was to clarify the molecular pathways through which the BvrR contributes to neuroinflammation and cognitive dysfunction. Human microglial clone 3 (HMC3) cells were transfected with pcDNA3.1‐BvrR‐His to examine the effects of BvrR from *Brucella abortus* on endoplasmic reticulum (ER) function and the activation of activating transcription factor 2 (ATF2) and nuclear factor kappa‐light‐chain‐enhancer of activated B cells (NF‐κB) p65. The role of phosphorylated inositol‐requiring enzyme 1 (p‐IRE1) in mediating BvrR‐induced activation of ATF2 and NF‐κB p65 was assessed by applying the IRE1 activator IXA4 and the IRE1 inhibitor GSK2850163, followed by evaluation with western blotting and RT‐qPCR. Interleukin‐6 (IL‐6) and tumor necrosis factor alpha (TNF‐α) concentrations in cell culture supernatants were quantified using ELISA. For in vivo analysis, HBAAV2/9‐IBA‐1‐BvrR‐6*HIS‐ZsGreen was stereotactically delivered into the right hippocampus of mice. Expression of BvrR in HMC3 cells induced phosphorylation of IRE1 and expansion of the ER. This activation enhanced ATF2 and NF‐κB p65 phosphorylation, facilitated their nuclear translocation, and significantly increased IL‐6 and TNF‐α expression at both the protein and mRNA levels. Inhibition of IRE1 with GSK2850163 suppressed these responses, whereas IRE1 activation with IXA4 reproduced the effects of BvrR. Findings indicate that BvrR from *B. abortus* activates IRE1, which subsequently stimulates ATF2 and NF‐κB p65, leading to increased expression of IL‐6 and TNF‐α and the induction of inflammatory responses in HMC3 cells.

## Graphical abstract

This study demonstrates that the BvrR induces neuroinflammation. In microglial cells, BvrR expression localizes to the endoplasmic reticulum (ER), causing ER expansion and activating the stress sensor IRE1. Phosphorylated IRE1 subsequently drives the activation and nuclear translocation of transcription factors ATF2 and NF‐κB p65. This signaling cascade leads to the upregulated transcription and secretion of pro‐inflammatory cytokines IL‐6 and TNF‐α. The pivotal role of IRE1 was confirmed, as its activation mimicked, and its inhibition blocked, BvrR's effects. In vivo, microglia‐specific expression of BvrR in the mouse hippocampus recapitulated this IRE1/ATF2/NF‐κB pathway, resulting in neuroinflammation, neuronal damage, and cognitive deficits, elucidating a key mechanism in neurobrucellosis.

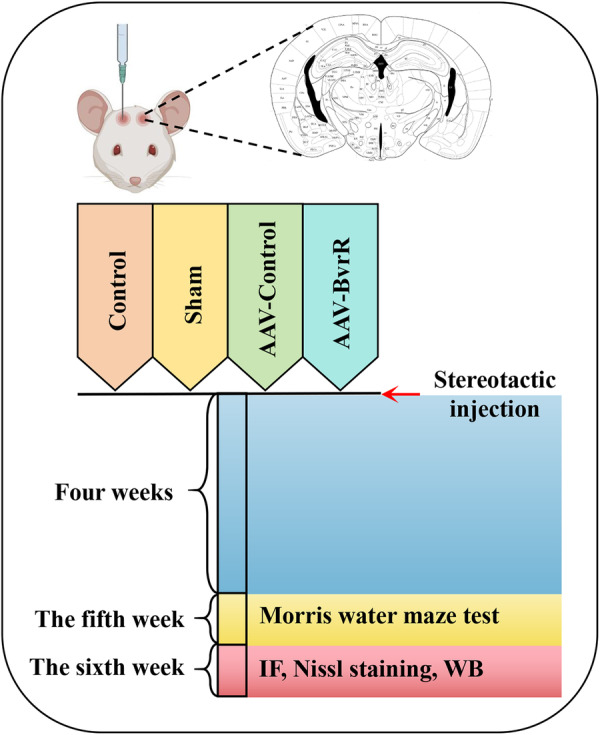

**Abbreviations**
AAV: adeno‐associated virus
ATF2: activating transcription factor 2
CNS: central nervous system
DAPI: 4′,6‐diamidino‐2‐phenylindole
ELISA: enzyme‐linked immunosorbent assay
ER: endoplasmic reticulum
HMC3: human microglial clone 3
MWM: Morris Water Maze
NF‐κB: nuclear factor kappa‐light‐chain‐enhancer of activated B cells
PBS: phosphate‐buffered saline
PFA: paraformaldehyde
p‐ATF2: phosphorylated ATF2
p‐p65: phosphorylated NF‐κB p65

## 1 Introduction

Neurobrucellosis, a severe complication of human brucellosis, occurs when *Brucella* invades the central nervous system (CNS) (Arazi et al. 2025; Zhuang et al. 2024). It is estimated to affect approximately 5–10% of individuals with brucellosis worldwide (Maji et al. 2020). Clinically, neurobrucellosis manifests with diverse neurological symptoms, including meningitis, encephalitis, and neuropsychiatric disorders, which may result in irreversible impairments in cognition and consciousness (Arazi et al. 2025; He et al. 2022; Soares et al. 2023). These deficits are closely associated with microglial activation and inflammation within the CNS following *Brucella* entry into the CNS (Rodríguez et al. 2017; Rebollada‐Merino et al. 2025). However, the underlying molecular mechanisms remain insufficiently defined. The present investigation aimed to clarify the molecular pathways contributing to *Brucella*‐induced neuroinflammation and their role in cognitive dysfunction.

*Brucella* spp. are facultative intracellular pathogens capable of invading macrophages and establishing chronic infections (Mallik et al. 2024; Guo et al. 2023). Microglia, the resident macrophages of the CNS, respond to such insults through immune activation and inflammatory signaling (De Vlaminck et al. 2022). The BvrR, a component of the essential BvrRS two‐component system, facilitates replication of *Brucella* within host endoplasmic reticulum (ER) compartments and modulates host immune responses, thereby contributing to bacterial persistence in neurobrucellosis (Rivas‐Solano et al. 2023; Altamirano‐Silva et al. 2021, 2023; Castillo‐Zeledón et al. 2023).

The ER stress sensor inositol‐requiring enzyme 1 (IRE1), activated in response to misfolded proteins, is critical for maintaining cellular homeostasis (Liu et al. 2024; Wiseman et al. 2022; Carreras‐Sureda et al. 2023). IRE1 activation regulates downstream signaling by activating transcription factor 2 (ATF2) and nuclear factor kappa‐light‐chain‐enhancer of activated B cells (NF‐κB) pathways (Zhang et al. 2022; Gendrisch et al. 2022). Phosphorylated ATF2 (p‐ATF2) controls interleukin‐6 (IL‐6) expression, while phosphorylated NF‐κB p65 (p‐p65) plays a central role in tumor necrosis factor alpha (TNF‐α) transcription (Zhao et al. 2023). The interaction between IRE1 and these inflammatory pathways underscores its importance in the pathogenesis of inflammatory diseases.

To date, there is no evidence on whether *Brucella* BvrR activates IRE1 to trigger ATF2 and NF‐κB p65 signaling. To address this knowledge gap, BvrR from *Brucella abortus* (a major human pathogen) was transfected into human microglial clone 3 (HMC3) cells, a widely used model for neuroinflammation (González‐Prieto et al. 2021). Findings indicate that BvrR activated IRE1, enhanced ATF2 and NF‐κB p65 phosphorylation, and elevated IL‐6 and TNF‐α levels. Moreover, hippocampal administration of BvrR in mice activated microglial p‐IRE1‐dependent inflammatory pathways, resulting in neuronal damage. These results provide mechanistic insight into the pathogenesis of neurobrucellosis and suggest potential therapeutic targets for anti‐inflammatory intervention.

## 2 Materials and Methods

### 2.1 Reagents, Antibodies, and Adeno‐Associated Virus (AAV)

The Whole Protein Extraction Kit (#20231218), BCA Protein Quantification Kit (#20231219), and SDS‐PAGE Reagent Kit (#20241206) were obtained from KeyGen Biotech (Nanjing, China). The Total RNA Extraction Kit (#A0308A) was provided by Tiangen Biotech (Beijing, China). The Transcriptor First Strand Complementary DNA (cDNA) Synthesis Kit (#91341024) was purchased from Thermo Fisher Scientific (Waltham, MA, USA). ChamQ SYBR qPCR Master Mix (#7E1581G4) was obtained from Vazyme Biotech (Nanjing, China). The IRE1 activator IXA4 (#145279) and inhibitor GSK2850163 (#40776) were acquired from MedChemExpress (New Jersey, USA). The Human IL‐6 Enzyme‐Linked Immunosorbent Assay (ELISA) Kit (#M241024‐004b) and Human TNF‐α ELISA Kit (#M241024‐102b) were purchased from NeoBioscience Technology (Shenzhen, China). ER‐Tracker Green (#C1042M‐1) was obtained from Beyotime Biotechnology (Shanghai, China). Primary antibodies against phosphorylated IRE1 (p‐IRE1; S724, #1002430‐2), p‐ATF2; T71 (#1023450‐12), and *GAPDH* (#GR3316865‐20) were sourced from Abcam (Cambridge, UK). Primary antibodies against IL‐6 (#66146‐1‐Ig) and TNF‐α (#60291‐1‐Ig) were obtained from Proteintech Group (Wuhan, China). The primary antibody against phosphorylated p65 (p‐p65; S536, #6100007483) was purchased from ABclonal Biotechnology (Wuhan, China). The adeno‐associated viruses HBAAV2/9‐IBA‐1‐ZsGreen (AAV‐Control) and HBAAV2/9‐IBA‐1‐BvrR‐6*HIS‐ZsGreen (AAV‐BvrR) were provided by Hanbio Biotechnology (Shanghai, China).

### 2.2 pcDNA3.1‐BvrR‐His and pcDNA3.1‐BvfA‐His Plasmids

The pcDNA3.1‐BvrR‐His and pcDNA3.1‐BvfA‐His plasmids (Figure S1) were obtained from Abiocenter Biotechnology. The *B. abortus bvrR* and *bvfA* gene sequences were retrieved from the National Center for Biotechnology Information (NCBI) database (Tables S1 and S2). Gene fragments were amplified by polymerase chain reaction (PCR) using specific primers (BvrR forward: 5′‐GCTAGCGTTTAAACTTAAGCTTGCCACCATGAAAGAGGCCTCTG‐3′, reverse: 5′‐AACGGGCCCTCTAGACTCGAGTCAGTGGTGGTGATGGTGGTGGGCTTCTCT‐3′; BvfA forward: 5′‐CTAGCGTTTAAACTTAAGCTTGCCACCATGGCCGAGGCCCAGG‐3′, reverse: 5′‐AACGGGCCCTCTAGACTCGAGTCAGTGGTGGTGGTGATGGTGCCGCTTGATG‐3′). The PCR conditions were as follows: initial denaturation at 94°C for 2 min; 30 cycles of 98°C for 10 s, 58°C for 10 s, and 68°C for 1 s; and a final extension at 68°C for 5 min. Both the amplified gene fragments and the pcDNA3.1 vector were digested with HindIII and XhoI. The digested products were ligated into the linearized vector using T4 DNA ligase at 50°C for 20 min. The ligation mixtures were then transformed into DH5α competent cells. Positive colonies were selected on ampicillin‐containing LB agar plates and verified by colony PCR. Recombinant plasmids were further confirmed by restriction enzyme analysis and sequencing prior to use in expression and transfection experiments with HMC3 cells.

### 2.3 AAV‐BvrR‐His Construct, Virus Production, Purification, and Titration

The AAV‐BvrR construct (Figure S2) was obtained from Hanbio Biotechnology. The *B. abortus bvrR* gene sequence (Table S1) was retrieved from the NCBI. Primers corresponding to the AAV vector cloning site were designed (forward: 5′‐TGATGCCTGGGAGTTAGCAAGGGATCCGCCACCATGAAAGAGGCCTCTG‐3′; reverse: 5′‐TTGGACTGGGCCATGGTGGCACCGGTAGGGCCGGGATTCTCCTCCAC‐3′). The gene fragment was amplified from cDNA using these primers with Phanta Super‐Fidelity DNA Polymerase under the following conditions: initial denaturation at 95°C for 5 min; 35 cycles of 95°C for 30 s, 60°C for 30 s, and 72°C for 1 min, followed by a final extension at 72°C for 10 min. The amplified product was gel‐purified, digested with XbaI and HindIII, and cloned into the AAV vector under the IBA‐1 promoter using HB Infusion (50°C for 30 min). The recombinant plasmid was transformed into *Escherichia coli* DH5α cells for propagation, extracted, and confirmed by sequencing. For viral production, AAV‐BvrR vectors were generated by co‐transfecting HEK293T cells with the AAV‐*bvrR* plasmid, pAAV‐RC, and pHelper plasmids using liposome‐mediated transfection. Viral particles were collected from the culture medium at 72 h post‐transfection by repeated freeze‐thaw cycles. Sterility and absence of mycoplasma contamination were verified. The viral titer (genome copies per milliliter) was determined by quantitative PCR (qPCR) using SYBR Green chemistry with WPRE‐specific primers (forward: 5′‐ACGCTATGTGGATACGCTGC‐3′; reverse: 5′‐CGGGCCACAACTCCTCATAA‐3′) against a WPRE plasmid standard curve.

### 2.4 Cell Culture

HMC3 cells (#AW‐CH0144; Anwei‐Sci Cell Center) were authenticated by short tandem repeat profiling and confirmed negative for mycoplasma contamination. Cells were maintained in 25 cm² flasks at a density of 1 × 10⁶ cells/flask in Dulbecco's Modified Eagle Medium supplemented with 10% fetal bovine serum and 1% penicillin‐streptomycin. Cultures were incubated at 37°C in a humidified atmosphere containing 5% CO₂. Cells were subcultured at a 1:3 ratio or harvested at approximately 80% confluence.

### 2.5 BvrR‐His Plasmid Transfection

HMC3 cells were seeded at 7 × 10⁵ cells/flask in 25 cm² flasks and cultured to ~60% confluence. A transfection complex was prepared by combining 10 μg pcDNA3.1‐BvrR‐His or control pcDNA3.1 plasmid with 500 μL Opti‐MEM and 25 μL Lipo8000 Transfection Reagent. The complex was added to cells, which were incubated at 37°C with 5% CO₂ for 4 days to allow for the expression of BvrR‐His prior to collection.

### 2.6 IXA4 Treatment In Vitro

A 1 mM IXA4 stock solution was prepared in DMSO and stored at −80°C. At 70% confluence, HMC3 cells were treated with 1.25, 2.50, 5.0, 10.0, or 20.0 μM IXA4 for 24 h. For inhibition experiments, pcDNA3.1‐BvrR‐His–transfected cells were treated with 10 μM IXA4 for 24 h before collection.

### 2.7 GSK2850163 Treatment In Vitro

A 1 mM GSK2850163 stock solution was prepared in anhydrous ethanol and stored at −80°C. HMC3 cells at 70% confluence were treated with 2.00, 4.00, 8.00, or 16.00 μM GSK2850163 for 24 h. For combination experiments, cells transfected with pcDNA3.1‐BvrR‐His were subsequently treated with 8 μM GSK2850163 for 24 h before collection.

### 2.8 Animals

Six‐week‐old male C57BL/6N specific pathogen‐free‐grade mice were obtained from Vital River Laboratory (Beijing, China). Animals were housed under controlled conditions (22°C–25°C, 50%–60% humidity, 12 h light/dark cycle) with ad libitum access to food and water. Following a 1‐week acclimatization, mice were randomly assigned to the following groups: Control, Sham, AAV‐control, and AAV‐*bvrR*. All animal procedures were approved by the Animal Care and Use Committee of the Air Force Medical University (Approval No. 20230770) and performed in accordance with national guidelines.

### 2.9 Stereotactic Injection (Intrahippocampal Injection)

Mice were anesthetized by intraperitoneal injection of 200 μL 3% sodium pentobarbital. Using stereotaxic coordinates relative to bregma (AP: −2.0 mm, ML: +1.5 mm, DV: −2.0 mm), 2.5 μL saline (Sham), AAV‐control, or AAV‐*bvrR* was injected into the right hippocampus at a rate of 0.75 μL/min with a Hamilton microsyringe (#1701). The injection needle was retained in place for 2 min post‐delivery to prevent backflow. Animals were recovered on a heating pad and maintained for 4 weeks before hippocampal tissue collection.

### 2.10 Western Blot Analysis

Total protein was extracted from HMC3 cells or hippocampal tissues using the Whole Protein Extraction Kit. Protein concentration was determined using the BCA assay. Equal amounts of protein (80 μg) were mixed with 5× loading buffer, denatured at 95°C for 10 min, separated on 8% to 12% SDS‐PAGE gels at 100 V for 2 h, and transferred onto polyvinylidene difluoride (PVDF) membranes (0.45 μm) at 300 mA. Membranes were blocked in 5% nonfat milk/TBST for 1 h and incubated with primary antibodies overnight at 4°C. After washing, membranes were incubated with IRDye 800CW goat anti‐rabbit IgG (H + L) for 1 h at room temperature (RT). Signals were visualized using the Odyssey Clx Imaging System, and band intensities were quantified relative to *GAPDH*.

### 2.11 ELISA

HMC3 culture supernatants were centrifuged (1000 rpm, 5 min, 4°C), and the clarified supernatants were collected for analysis. Protein concentrations of TNF‐α and IL‐6 were quantified using human TNF‐α and IL‐6 ELISA kits according to the manufacturer's instructions. Absorbance was measured at 450 nm with a TECAN Infinite 200Pro microplate reader.

### 2.12 RT‐qPCR Assay

HMC3 cells were seeded at 4 × 10⁵ cells per 6‐cm dish and treated at 65% confluence. Total RNA was extracted and reverse‐transcribed into cDNA. RT‐qPCR was conducted using ChamQ SYBR qPCR Master Mix on a LightCycler 480 II system with the following primers (Sangon Biotech): *IL6*: Fwd 5′‐GGTGTTGCCTGCTGCCTTCC‐3′, Rev 5′‐GTTCTGAAGAGGTGAGTGGCTGTC‐3′; *TNF*: Fwd 5′‐TGGCGTGGAGCTGAGAGATAACC‐3′, Rev 5′‐CGATGCGGCTGATGGTGTGG‐3′; *GAPDH*: Fwd 5′‐CAAGGTCATCCATGACAACTTTG‐3′, Rev 5′‐GTCCACCACCCTGTTGCTGTAG‐3′. The amplification protocol was: 95°C for 30 s, followed by 40 cycles of 95°C for 10 s, 56°C for 30 s, and 72°C for 30 s, with melting curve analysis at 95°C for 15 s, 60°C for 60 s, and 95°C for 15 s. Relative mRNA expression of IL‐6 and TNF was calculated using the 2^−ΔΔCt^ method with *GAPDH* as the reference gene. All reactions were performed in triplicate with at least four biological replicates.

### 2.13 Transmission Electron Microscopy Assay

Following treatment, HMC3 cells (1 × 10⁶) were washed with cold phosphate‐buffered saline (PBS) and fixed on ice for 15 min with 3 mL cold fixative. Cells were scraped and stored in fixative at 4°C overnight. Samples were centrifuged, embedded in 1% agarose, and post‐fixed with 1% osmium tetroxide for 2 h at RT in the dark. After three phosphate‐buffer washes (15 min each), samples were dehydrated through a graded ethanol series (30%–100%, 20 min each), followed by two 15‐min acetone washes. Infiltration with embedding resin was performed overnight at 37°C, followed by polymerization at 60°C for 48 h. Ultrathin sections (60–80 nm) were cut, mounted on copper grids, stained with 2% uranyl acetate (8 min), rinsed, stained with 2.6% lead citrate (8 min), and air‐dried overnight. Sections were examined with a Hitachi HT7800 TEM at 100 kV (point‐to‐point resolution: 0.2 nm).

### 2.14 Immunofluorescent Assay

#### 2.14.1 Cell Immunofluorescence

HMC3 cells (1 × 10⁵/well) were seeded onto coverslips placed in 12‐well plates. Following treatment, the cells were washed three times with cold PBS, fixed with 4% paraformaldehyde (PFA) for 20 min on ice, and permeabilized using 0.3% Triton X‐100 in PBS for 10 min at RT. The cells were blocked with 10% goat serum for 1 h at RT and incubated overnight at 4°C with primary antibodies against p‐ATF2 and p‐p65 (1:200). After PBS washes, the cells were incubated with Alexa Fluor 555 (#4413S) and Alexa Fluor 488 (#2668665) secondary antibodies for 1 h at 37°C in the dark. Nuclei were counterstained with 4′,6‐diamidino‐2‐phenylindole (DAPI) for 10 min at RT. Coverslips were mounted and imaged using a confocal microscope (FV3000, Olympus, Tokyo, Japan).

#### 2.14.2 Immunofluorescence of Brain Sections

Mice were deeply anesthetized with an intraperitoneal injection of 200 μL 3% sodium pentobarbital and transcardially perfused with 20 mL cold 4% PFA followed by saline. Brains were post‐fixed in 4% PFA for 24 h, cryoprotected in 25% sucrose for 48 h, embedded in optimal cutting temperature (OCT) compound, and coronally sectioned at 10 μm. Sections were permeabilized with 0.3% Triton X‐100 in PBS for 15 min, blocked with 5% goat serum for 1 h at RT, and incubated overnight at 4°C with primary antibodies against ionized calcium‐binding adapter molecule 1 (IBA‐1), p‐IRE1, p‐ATF2, or p‐p65 (1:200). After PBS washes, sections were incubated with Alexa Fluor 555 and Alexa Fluor 647 (#2577247) secondary antibodies for 1 h at 37°C in the dark. Nuclei were counterstained with DAPI for 10 min, mounted, and imaged using a confocal microscope.

### 2.15 Morris Water Maze (MWM)

The MWM consisted of a circular pool (120 cm in diameter, 40 cm deep) filled with water maintained at 25°C and rendered opaque with kaolin powder. A hidden escape platform (10 cm in diameter) was submerged 1 cm below the water surface within one quadrant. Mice were acclimatized to the experimental room 1 day before testing. Training was conducted over 4 consecutive days, with four trials per day and an inter‐trial interval of at least 30 min. Mice were released from randomly assigned quadrants. If the platform was not located within 60 s, mice were guided to it and allowed to remain there for 30 s. On Day 5 (probe trial), the platform was removed. Mice were released from the quadrant opposite to the target, and the following parameters were recorded during a 1‐min trial: time spent in the target quadrant, swimming distance within the target quadrant, and the number of accurate crossings at the previous platform location.

### 2.16 Nissl Staining

Brains were removed and postfixed as described in Section 2.14.2. Fixed brains were dehydrated through graded ethanol solutions (70%, 80%, 90%, and 100%) and cleared in xylene for 30 min. Samples were subsequently embedded in paraffin wax, cooled, and solidified at RT. Paraffin blocks were trimmed to isolate the hippocampal region, and coronal sections (7 µm) were prepared using a rotary microtome. Sections were deparaffinized in xylene (two times, 5 min each), rehydrated through descending ethanol series (100% to 70%, 5 min each), stained with 0.1% cresyl violet for 15 min at RT, rinsed in distilled water, and differentiated in 95% ethanol for 30 s. Sections were then dehydrated in 100% ethanol, cleared in xylene (5 min), and mounted with neutral resin. Images were obtained using an OLYMPUS VS200 microscope, and neuronal quantification was performed with ImageJ software.

### 2.17 Statistical Analysis

All experiments included at least three biological replicates. Data were expressed as mean ± standard deviation. Statistical analyses were conducted using GraphPad Prism version 9.5.1. Normality of distributions was assessed with the Shapiro–Wilk test, and homogeneity of variance was evaluated with the Brown‐Forsythe test. Group comparisons were performed using one‐way analysis of variance, followed by Dunnett's T3 post hoc test. A *p*‐value ≤ 0.05 was considered statistically significant.

## 3 Results

### 3.1 BvrR Activates IRE1 and Causes Expansion of the ER Membrane

**Figure 1:**
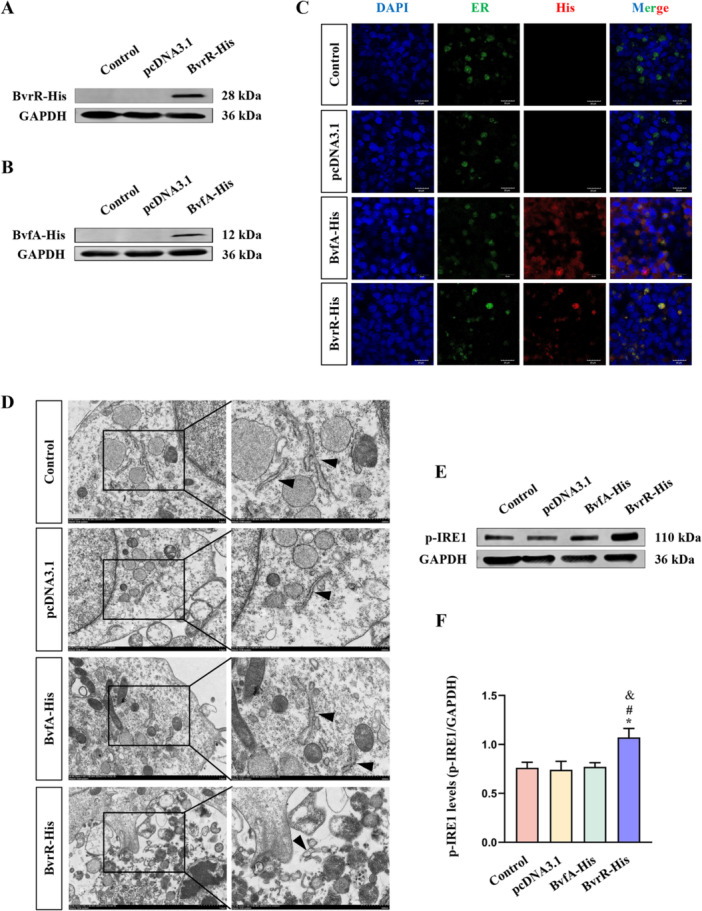
Effect of *Brucella* BvrR protein on IRE1 activation and ER morphology in HMC3 cells. (A) Western blot analysis of BvrR‐His protein expression. (B) Western blot analysis of BvfA‐His protein expression. (C) Subcellular localization of BvrR‐His and ER in HMC3 cells observed by laser confocal microscopy. (D) Transmission electron microscopy analysis of ER morphology in HMC3 cells from each group. (E) Western blot analysis of p‐IRE1 protein levels. (F) Quantification of p‐IRE1 protein levels normalized to GAPDH. Data are presented as means ± standard deviations from three independent replicates; *p < 0.05 versus Control; ^#^ *p* < 0.05 versus pcDNA3.1; ^&^ *p* < 0.05 versus BvfA‐His.

To assess BvrR‐mediated activation of IRE1 and the associated expansion of the ER, HMC3 cells were transfected with the pcDNA3.1‐BvrR‐His plasmid. Western blot analysis confirmed the expression of BvrR‐His and BvfA‐His proteins in their respective transfected groups, while no detectable expression was observed in either the pcDNA3.1 or Control groups (Figure 1A,B). Because BvrR participates in ER‐dependent replication of *Brucella*, the subcellular localization of BvrR‐His was examined by immunofluorescence staining (Altamirano‐Silva et al. 2023). BvrR‐His protein was detected in the ER of HMC3 cells in the BvrR‐His group (Figure 1C). In contrast, BvfA‐His protein was localized in both the ER and cytoplasm in the BvfA‐His group (Figure 1C). Transmission electron microscopy (TEM) was performed to evaluate the effect of BvrR‐His and BvfA‐His expression on ER morphology. Marked ER dilation was observed in the BvrR‐His group, whereas ER morphology remained unchanged in the other three groups (Figure 1D). Western blot analysis was further conducted to assess p‐IRE1 levels (Figure 1E). Protein levels of p‐IRE1 were significantly higher in the BvrR‐His group compared with the other three groups (Figure 1F). Collectively, these findings indicate that expression of the BvrR activated IRE1 in HMC3 cells and contributed to ER dilation.

### 3.2 BvrR Promotes ATF2 and NF‐κB p65 Activation

**Figure 2:**
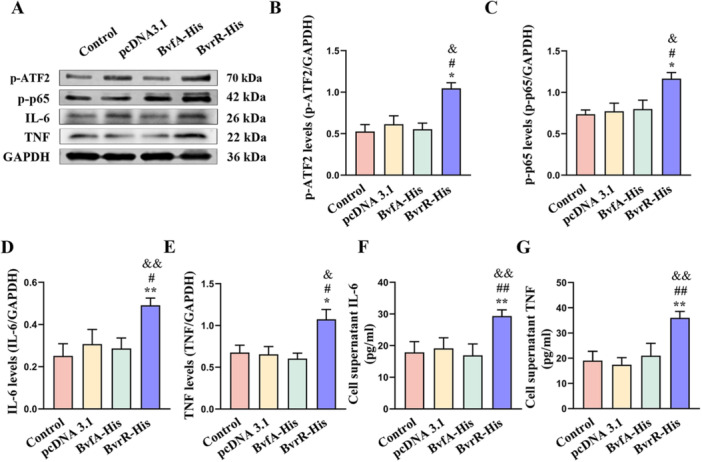
*Brucella* BvrR increases p‐ATF2, p‐p65, IL‐6, and TNF‐α protein levels in HMC3 cells. (A) Western blot analysis of p‐ATF2, p‐p65, IL‐6, and TNF‐α. (B–E) Quantification of p‐ATF2, p‐p65, IL‐6, and TNF‐α levels normalized to GAPDH. (F and G) ELISA measurement of IL‐6 and TNF‐α levels in culture media. Data are presented as means ± standard deviations from three independent experiments; *p < 0.05, **p < 0.01 versus Control; ^#^ *p* < 0.05, ^##^ *p* < 0.01 versus pcDNA3.1; ^&^ *p* < 0.05, ^&&^ *p* < 0.01 versus BvfA‐His.

Because p‐IRE1 can activate ATF2 and NF‐κB p65 proteins, the p‐ATF2, p‐p65, IL‐6, and TNF‐α protein levels were assessed by western blot (Figure 2A) (Zhang et al. 2022; Gendrisch et al. 2022). Protein levels of p‐ATF2, p‐p65, IL‐6, and TNF‐α were significantly elevated in the BvrR‐His group compared with the other three groups (Figure 2B–E). ELISA further demonstrated that IL‐6 and TNF‐α concentrations were significantly higher in the BvrR‐His group relative to the other three groups (Figure 2F,G), consistent with the western blot findings (Figure 2D,E). These results indicated that the BvrR promoted the expression of IL‐6 and TNF‐α through activation of ATF2 and NF‐κB p65 proteins.

### 3.3 BvrR Enhances Nuclear Translocation of p‐ATF2 and p‐p65

**Figure 3:**
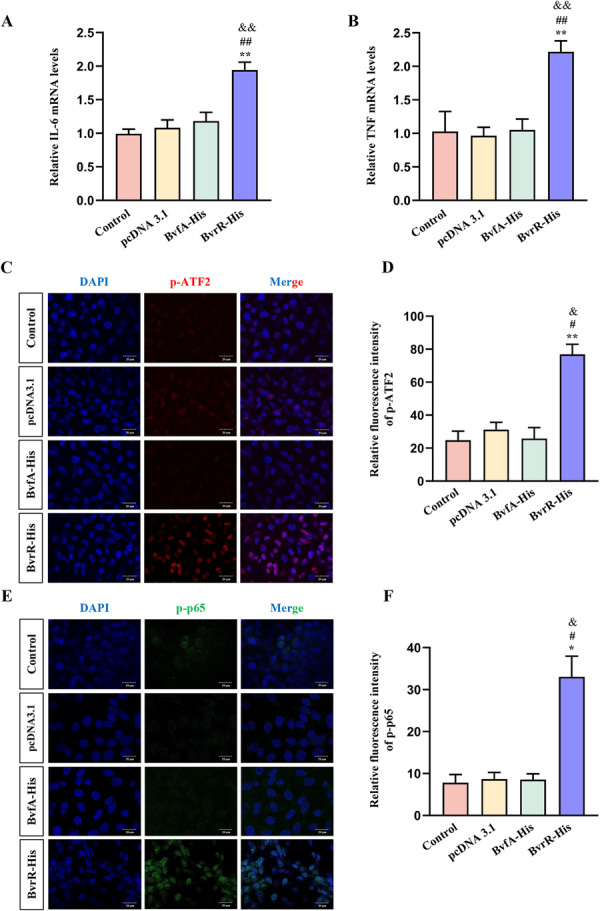
*Brucella* BvrR enhances nuclear translocation of p‐ATF2 and p‐p65. (A) RT‐qPCR analysis of IL‐6 mRNA expression. (B) RT‐qPCR analysis of TNF mRNA expression. (C) Immunofluorescence analysis of p‐ATF2 nuclear translocation. (D) Quantification of p‐ATF2 fluorescence intensity. (E) Immunofluorescence analysis of p‐p65 nuclear translocation in HMC3 cells. (F) Quantification of p‐p65 fluorescence intensity. Data are presented as means ± standard deviations from three independent experiments; *p < 0.05, **p < 0.01 versus Control; ^#^ *p* < 0.05, ^##^ *p* < 0.01 versus pcDNA3.1; ^&^ *p* < 0.05, ^&&^ *p* < 0.01 versus BvfA‐His.

p‐ATF2 and phosphorylated p65 function as transcription factors that enhance *IL6* and *TNF* transcription upon nuclear translocation (Han et al. 2020; Zhao et al. 2024). RT‐qPCR demonstrated significantly increased mRNA expression of *IL6* and *TNF* in the BvrR‐His group compared with the other three groups (Figure 3A,B). To further assess nuclear translocation, immunofluorescence staining was performed. Nuclear localization of both p‐ATF2 (Figure 3C,D) and p‐p65 (Figure 3E,F) was significantly higher in the BvrR‐His group compared with the other three groups. These findings indicate that BvrR enhanced the nuclear translocation of p‐ATF2 and p‐p65, thereby promoting *IL6* and *TNF* expression at the transcriptional level.

### 3.4 Activation of ATF2 and NF‐κB p65 Proteins Mediated by BvrR‐Induced IRE1 Signaling

**Figure 4:**
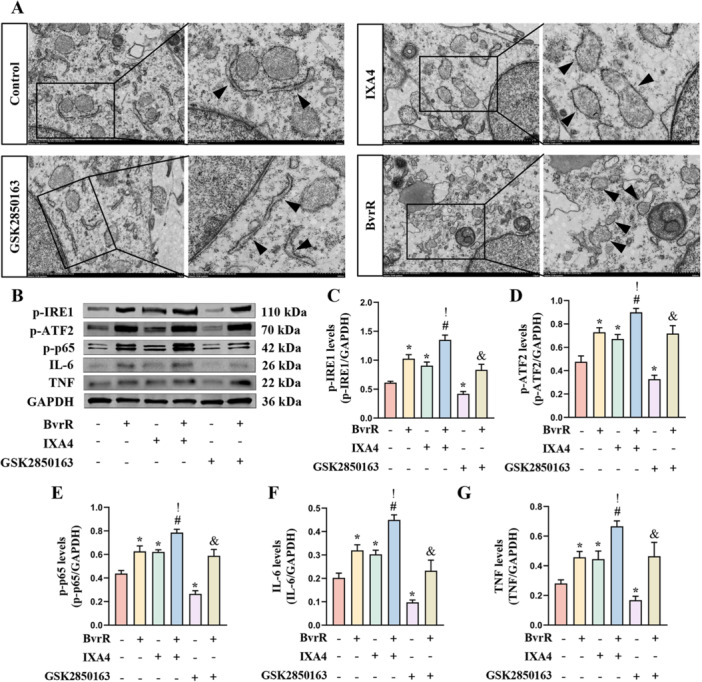
*Brucella* BvrR activates ATF2/IL‐6 and NF‐κB p65/TNF‐α signaling via IRE1. (A) Transmission electron microscopy analysis of ER morphology in HMC3 cells. (B) Western blot analysis of p‐IRE1, p‐ATF2, p‐p65, IL‐6, and TNF‐α protein levels. (C–G) Quantification of p‐IRE1, p‐ATF2, p‐p65, IL‐6, and TNF‐α expression normalized to GAPDH. Data are presented as means ± standard deviations from at least three independent experiments; *p < 0.05 versus Control; ^#^ *p* < 0.05 versus IXA4; ^&^ *p* < 0.05 versus GSK2850163; ^!^ *p* < 0.05 versus BvrR.

The effects of 10 μM IXA4 (Figure S3), 8 μM GSK2850163 (Figure S4), and BvrR on the ER in HMC3 cells were further examined. TEM was used to evaluate ER morphology in each group. Marked dilation of the ER was observed in both the IXA4 and BvrR groups, whereas no notable morphological alterations were detected between the control or GSK2850163 groups (Figure 4A). To assess whether BvrR could activate ATF2 and NF‐κB p65 through p‐IRE1, protein expression levels of p‐IRE1, p‐ATF2, p‐p65, IL‐6, and TNF‐α were measured by western blotting (Figure 4B). Compared with the control group, significant increases in these protein levels were identified in the BvrR and IXA4 groups, whereas the GSK2850163 group demonstrated significant reductions (Figure 4C–G). Protein expression in the BvrR+IXA4 and BvrR+GSK2850163 groups was significantly elevated relative to their respective control groups (Figure 4C–G). Furthermore, protein levels in the BvrR+IXA4 group were significantly higher than those in the BvrR group, while no statistically significant difference was observed between the BvrR+GSK2850163 and BvrR groups (Figure 4C–G).

**Figure 5:**
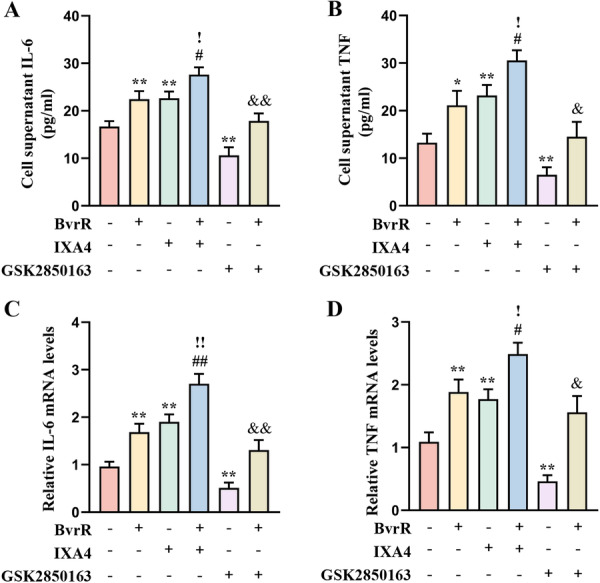
Effect of BvrR, IXA4, and GSK2850163 on IL‐6 and TNF expression. (A) ELISA measurement of IL‐6 in supernatants. (B) ELISA measurement of TNF‐α in supernatants. (C) RT‐qPCR analysis of *IL6* mRNA expression. (D) RT‐qPCR analysis of *TNF* mRNA expression. Each assay was performed in six replicates, and data are presented as means ± standard deviations; *p < 0.05, **p < 0.01 versus Control; ^#^ *p* < 0.05, ^##^ *p* < 0.01 versus IXA4; ^&^ *p* < 0.05, ^&&^ *p* < 0.01 versus GSK2850163; ^!^ *p* < 0.05, ^!!^ *p* < 0.01 versus BvrR.

Subsequent ELISA assays were performed to quantify IL‐6 and TNF‐α in the cell supernatants. Concentrations of both cytokines were significantly increased in the BvrR and IXA4 groups and markedly reduced in the GSK2850163 group compared with the Control group (Figure 5A,B). Pro‐inflammatory cytokine levels were significantly elevated in both the BvrR+IXA4 and BvrR+GSK2850163 groups compared with their corresponding Control groups (Figure 5A,B). Notably, cytokine concentrations in the BvrR+IXA4 group were significantly higher than in the BvrR group, whereas no significant difference was identified between the BvrR+GSK2850163 and BvrR groups (Figure 5A,B). Finally, relative mRNA expression levels of *IL‐6* and *TNF*, assessed by RT‐qPCR, were consistent with the ELISA findings (Figure 5C,D). Collectively, these results indicate that *Brucella* BvrR may promote IRE1 activation, which subsequently enhances ATF2 and NF‐κB p65 signaling, leading to upregulation of IL‐6 and TNF‐α expression.

### 3.5 Effects of the BvrR Protein on the Hippocampus Neurons in C57BL/6N Mice

**Figure 6:**
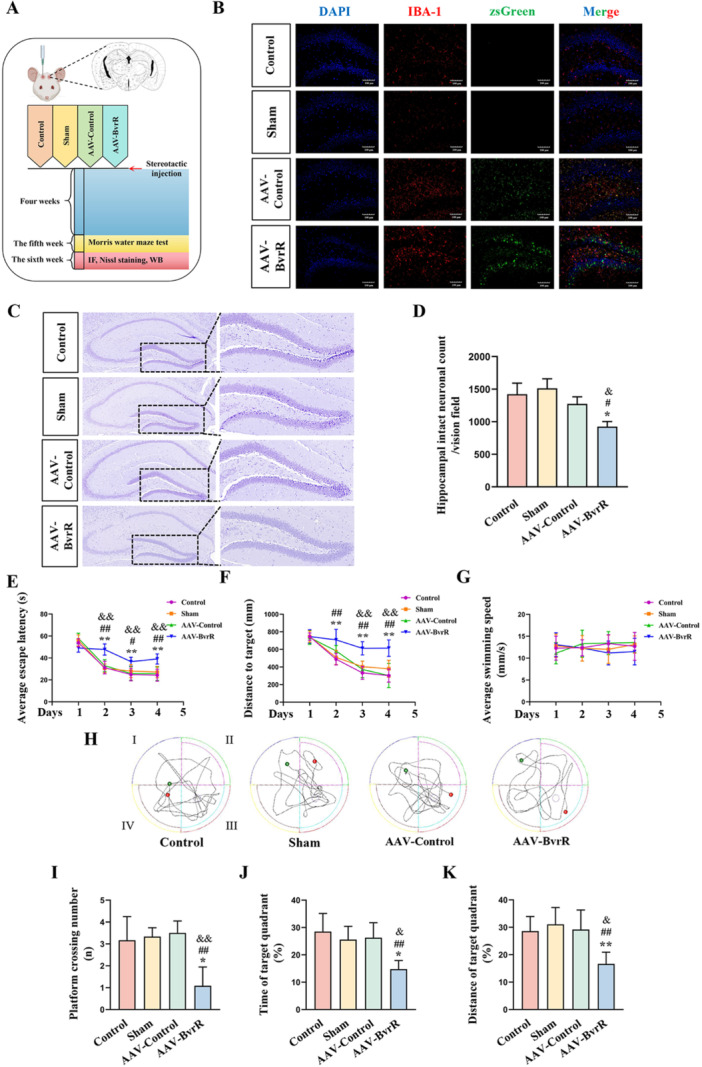
Influence of BvrR protein on hippocampal neurons in C57BL/6N mice. (A) Flow diagram of the experimental design. (B) Immunofluorescence staining of IBA‐1 and zsGreen in hippocampal tissue. (C) Nissl staining of hippocampal neurons. (D) Quantification of intact neurons in the hippocampus (*n* = 3/group). (E) Escape latency during 4 days of training. (F) Distance to target during 4 days of training. (G) Swimming speed during 4 days of training. (H) Representative swimming paths on Day 5. (I) Platform crossing number on Day 5. (J) Percentage of time spent in the target quadrant on Day 5. (K) Percentage of distance traveled in the target quadrant on Day 5. Data were obtained from six mice per group and are presented as means ± standard deviations; *p < 0.05, **p < 0.01 versus Control; ^#^ *p* < 0.05, ^##^ *p* < 0.01 versus Sham; ^&^ *p* < 0.05, ^&&^ *p* < 0.01 versus AAV‐Control.

*Brucella* BvrR has been associated with microglia‐mediated inflammation. To evaluate its impact, BvrR was expressed in microglia within the right hippocampus of C57BL/6N mice for 4 weeks through stereotaxic injection of AAV serotype 2/9 vectors encoding BvrR (AAV‐BvrR). Mice receiving empty recombinant AAV serotype 2/9 vectors served as controls (AAV‐Control) (Figure 6A). Immunofluorescence analysis of zsGreen and IBA‐1 in the hippocampus confirmed microglial transfection in both AAV‐BvrR and AAV‐Control groups, as evidenced by the co‐localization of ZsGreen protein with IBA‐1 protein (Figure 6B).

Neuronal survival was evaluated using Nissl staining, with Nissl bodies serving as markers of neuronal viability (Liu, Zhou, et al. 2022). Compared to the other three groups, hippocampal neurons in the AAV‐BvRr group showed significantly lighter staining, indicating chromatin dissolution in hippocampal neurons and suggesting reversible damage (Figure 6C,D). The hippocampus, a critical region involved in learning, memory, and emotional regulation, was further examined for cognitive function using the MWM test (Liu, Wu, et al. 2022). From Days 2 to 4, mice in the AAV‐*bvrR* group demonstrated significantly longer average escape latency and greater distance traveled to the target compared with the other groups (Figure 6E,F), with no significant group differences in average swimming speed (Figure 6G). On Day 5, the AAV‐*bvrR* group exhibited a significantly lower platform crossing number (Figure 6H,I), as well as reduced percentage of time and distance in the target quadrant (Figure 6J,K). These findings indicate that *Brucella* BvrR expression induced reversible neuronal damage in the hippocampus and impaired learning and memory performance in C57BL/6N mice.

### 3.6 *Brucella* BvrR Induces Activation of ATF2 and NF‐κB p65 in Hippocampal Microglia via p‐IRE1

**Figure 7:**
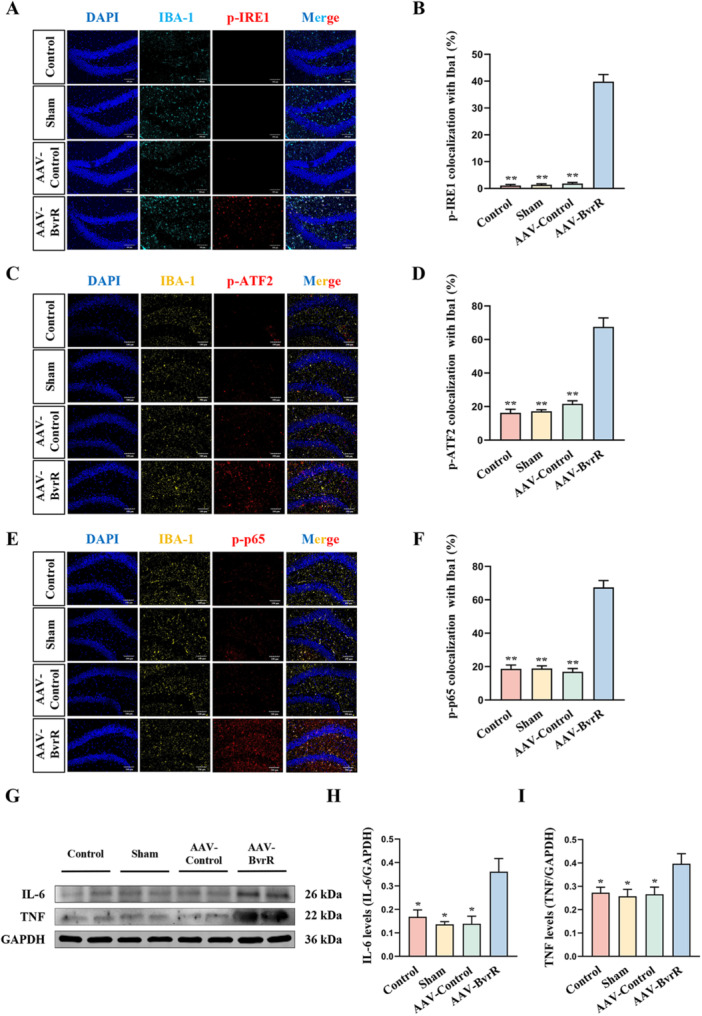
*Brucella* BvrR activates IRE1, ATF2, and NF‐κB p65 in hippocampal microglia. (A) Immunofluorescence co‐localization of IBA‐1 and p‐IRE1. (B) Quantification of p‐IRE1 co‐localization with IBA‐1. (C) Immunofluorescence co‐localization of IBA‐1 and p‐ATF2. (D) Quantification of p‐ATF2 co‐localization with IBA‐1. (E) Immunofluorescence co‐localization of IBA‐1 and p‐p65. (F) Quantification of p‐p65 co‐localization with IBA‐1. (G) Western blot analysis of IL‐6 and TNF‐α protein levels in hippocampal tissue. (H) Quantification of IL‐6 expression normalized to GAPDH. (I) Quantification of TNF‐α expression normalized to GAPDH. Data are presented as means ± standard deviations from three independent experiments; *p < 0.05, **p < 0.01 versus AAV‐*bvrR*.

To investigate whether *Brucella* BvrR activated ATF2 and NF‐κB p65 through IRE1 signaling, immunofluorescence staining was performed to assess the co‐localization of p‐IRE1, p‐ATF2, and p‐p65 with IBA‐1 in the hippocampus of mice. The findings indicated that p‐IRE1 co‐localization with IBA‐1 was significantly higher in the AAV‐BvrR group compared with the other groups (Figure 7A,B). Similarly, co‐localization of p‐ATF2 (Figure 7C,D) and p‐p65 (Figure 7E,F) with IBA‐1 was markedly elevated in the AAV‐*bvrR* group.

Western blot analysis was subsequently used to measure the protein levels of IL‐6 and TNF‐α in the hippocampus (Figure 7G). Both IL‐6 and TNF‐α levels were significantly increased in the AAV‐*bvrR* group compared with the other three groups (Figure 7H,I). Collectively, these results indicate that BvrR‐mediated IRE1 activation promoted downstream activation of ATF2 and NF‐κB p65 in hippocampal microglia, which was associated with increased IL‐6 and TNF‐α expression, thereby contributing to neuronal injury.

## 4 Discussion

In neurobrucellosis, *Brucella* causes CNS infection, specifically targeting microglia (Rodríguez et al. 2017). After infecting microglia, *Brucella* interacts with the ER to establish a specialized replicative niche (Miller et al. 2017). ER stress induced by *Brucella* infection can trigger the unfolded protein response (UPR), which exacerbates neuroinflammation and contributes to neuronal damage, synaptic dysfunction, and cognitive impairment (Arazi et al. 2025; Luizet et al. 2021; Wang et al. 2025; Esmael et al. 2021). The neuroinflammatory response to *Brucella* infection has been closely associated with its virulence factors, particularly BvrR (Altamirano‐Silva et al. 2021). Findings from this study indicate that *B. abortus* BvrR activated IRE1, which subsequently triggered the ATF2 and NF‐κB p65 proteins, leading to inflammatory responses in HMC3 microglia. In vivo experiments supported the in vitro findings, confirming that *B. abortus* BvrR‐induced microglial inflammation resulted in hippocampal neuronal damage, impairing learning and memory functions.

In the present study, HMC3 cells were transfected with BvrR to evaluate its ER co‐localization and the expansion of the ER membrane‐inducing effects. BvrR plays a central role in *Brucella* replication within the ER (Altamirano‐Silva et al. 2021; Głowacka et al. 2018). *Brucella* regulates ER stress and dynamics to facilitate replication, and the observed interaction between BvrR and the ER underscores the importance of this virulence factor in this process (Zhi et al. 2019). Consistent with prior findings, the current results indicate that BvrR co‐localized with the ER and promoted its expansion, reinforcing its role in the intracellular lifecycle of *Brucella*. IRE1, a key ER stress sensor, is critical for the initiation of the UPR. Notably, Guimarães et al. reported that *B. abortus* infection activated IRE1 in macrophages, exploiting this stress response pathway to enhance intracellular survival (Guimarães et al. 2023). These findings support the current observation that *B. abortus* BvrR‐induced phosphorylation and subsequent activation of IRE1 in HMC3 cells.

*B. abortus* not only activated IRE1 but also induced inflammation in microglia. Rodríguez et al. reported that *B. abortus* infection activated microglia, resulting in increased production of IL‐6 and TNF‐α, both of which are central mediators of neuroinflammation (Rodríguez et al. 2024). In the present study, IL‐6 and TNF‐α levels were measured in both cells and supernatants, confirming that *B. abortus* BvrR induced these cytokines in HMC3 cells. However, unlike previous findings, inflammation was triggered through intracellular expression of BvrR rather than direct infection (Rodríguez et al. 2024). ATF2 and NF‐κB p65 are transcription factors that regulate inflammation by promoting the expression of pro‐inflammatory cytokines, such as IL‐6 and TNF‐α (Otsuki et al. 2021). The results confirmed that elevated IL‐6 and TNF‐α levels were attributable to BvrR‐induced activation of ATF2 and NF‐κB p65, along with enhanced nuclear translocation. These findings are consistent with existing evidence but uniquely identify BvrR as a virulence factor responsible for inducing microglial inflammation.

IRE1 activation initiated the phosphorylation of ATF2 and NF‐κB p65, thereby promoting pro‐inflammatory cytokine expression and underscoring IRE1's central role in regulating inflammation (Zhang et al. 2022; Zha et al. 2015). To examine whether BvrR activated ATF2 and NF‐κB p65 through p‐IRE1, HMC3 cells were treated with varying concentrations of the IRE1 activator IXA4 and the inhibitor GSK2850163. The findings indicated that 10 μM IXA4 activated IRE1, ATF2, and NF‐κB p65, which subsequently enhanced IL‐6 and TNF‐α expression, whereas 8 μM GSK2850163 exerted an antagonistic effect. This suggests that ATF2 and NF‐κB p65 activation were dependent on IRE1 activity. These observations are consistent with those of Hsieh et al. who reported that LPS treatment of A549 cells induced IRE1 phosphorylation, activated NF‐κB p65, and increased TNF‐α expression (Zhang et al. 2022; Hsieh et al. 2022). In contrast, the findings of Lyu et al. differed, as suppression of IRE1 in cancer cells from patients with oropharyngeal cancer markedly reduced IL‐6 expression induced by radiation (Lyu et al. 2019). Such discrepancies may be attributable to differences in cell sources and methods of inflammation induction, as the present study employed BvrR plasmid transfection, whereas theirs involved radiation‐induced inflammation.

Building on previous research, the present study investigated the activation of IRE1 by *B. abortus* BvrR and its subsequent impact on the activation of ATF2 and NF‐κB p65 proteins. The modulation of host inflammatory responses by BvrR through the ER sensor protein IRE1 has been identified as an important area of research, with several notable findings. Keestra‐Gounder et al. reported that the *B. abortus* virulence factor VceC activated IRE1, which in turn activated NF‐κB p65, resulting in the upregulation of *IL6* expression and the induction of inflammatory responses in murine macrophages (Keestra‐Gounder et al. 2016). In contrast, Li et al. found that exogenous expression of the *B. abortus* virulence factor BspI in HeLa cells inhibited IRE1 activation, which suppressed NF‐κB p65 activation and reduced *IL6* and *TNF* expression (Li et al. 2022).

The present findings indicate that *B. abortus* BvrR activated IRE1, which subsequently activated ATF2 and NF‐κB p65, thereby enhancing IL6 and TNF expression and inducing inflammation in HMC3 cells. These results were consistent with those of Keestra‐Gounder et al. supporting the view that BvrR elicit host inflammatory responses via IRE1 activation (Keestra‐Gounder et al. 2016). However, no significant differences in p‐IRE1, p‐ATF2, p‐p65, IL6, and TNF‐α levels were observed between the BvrR and BvrR+GSK2850163 groups. This lack of difference may be attributable to the intracellular expression of BvrR, which likely impeded the binding of GSK2850163 to the p‐IRE1 site, thereby resulting in unaltered protein levels.

The discrepancies between the present findings and those of previous reports may reflect the distinct regulatory effects exerted by different *B. abortus* virulence factors on host inflammatory responses, as well as the differential sensitivities of various cell lines to these factors.

Moreover, the findings indicated that activation of IRE1 by either BvrR or IXA4 induced swelling and expansion of the ER in HMC3 cells, consistent with the results reported by Ouyang et al. (2019). This effect may have resulted from excessive accumulation of exogenously expressed BvrR within the ER, which likely promoted overactivation of IRE1, thereby inducing marked ER stress and subsequent expansion of the ER membrane.

In conclusion, the present study confirmed that *B. abortus* BvrR activated ATF2 and NF‐κB p65 through IRE1 signaling, thereby driving an inflammatory response in HMC3 cells. To further substantiate these findings, in vivo experiments were performed. The most severe manifestations of neurobrucellosis, such as psychiatric symptoms and cognitive disturbances, have been reported previously (Esmael et al. 2021). In the current study, *B. abortus* BvrR‐induced hippocampal neurons reversible damage in C57BL/6N mice, which resulted in impairments in learning, memory, and cognitive functions. These results were consistent with the findings of Fathi et al. who reported that *B. abortus* 544 activated microglia in the hippocampus of albino rats, leading to inflammation and subsequent neuronal damage (Fathi et al. 2024).

To corroborate the in vitro findings, immunofluorescence analysis was performed to assess the co‐localization of p‐IRE1, p‐ATF2, and p‐p65 with IBA‐1. The results demonstrated that *B. abortus* BvrR activated IRE1, ATF2, and NF‐κB p65 in hippocampal microglia, which supported the inflammatory mechanism observed in vitro. Overall, the in vivo findings indicate that *B. abortus* BvrR activated IRE1 in microglia, subsequently inducing ATF2 and NF‐κB p65 activation, thereby enhancing IL‐6 and TNF‐α expression and resulting in neuronal damage within the hippocampus.

A key limitation of this study was the inability to construct a *bvrR* gene knockout in *B. abortus*, primarily due to restricted access to a Biosafety Level 3 (BSL‐3) laboratory, which is required for research involving live bacteria. To address this limitation, future investigations will aim to collaborate with BSL‐3 facilities to enable infection of microglia or mice with *bvrR* knockout *B. abortus*, thereby providing further insight into the role of BvrR in neurobrucellosis. Additionally, the mechanisms through which BvrR activates the IRE1 ER sensor were not explored in this study. Future research will focus on detailed investigations to elucidate these mechanisms and further clarify the biological functions of BvrR in *B. abortus*.

## 5 Conclusion

This study demonstrated that BvrR from *B. abortus* activated the IRE1 protein, which subsequently stimulated ATF2 and NF‐κB p65, resulting in increased expression of IL‐6 and TNF‐α and triggering inflammatory responses in HMC3 cells. The findings partially elucidated the mechanism through which *B. abortus* BvrR induces microglial inflammation. Targeting key proteins in this signaling pathway may offer potential therapeutic strategies for mitigating *Brucella*‐associated neuroinflammation. Furthermore, these results provide experimental evidence that supports future clinical translation for the treatment of neurobrucellosis.

## Author Contributions

**Zhao Wang:** conceptualization, validation, data curation, writing – original draft preparation, writing – review and editing, funding acquisition. **Xinwen Yu:** conceptualization, validation, writing – review and editing. **Boyu Liu:** methodology, validation, data curation. **Dongni Ren:** methodology, validation.

## Ethics Statement

All animal procedures received approval from the Animal Care and Use Committee of the Air Force Medical University (Approval No. 20230770; date of approval: March 08, 2023).

## Consent

The authors have nothing to report.

## Conflicts of Interest

The authors declare no conflicts of interest.

## Supporting information


**Figure S1:** Vector maps.

**Figure S2:** AAV‐BvrR Vector map.

**Figure S3:** The role of IXA4 in activating IRE1 and triggering inflammatory pathways in HMC3 cells.

**Figure S4:** Inhibition of IRE1, ATF2, and NF‐κB p65 activation by GSK2850163.

**Table S1:** BvrR sequence information. **Table S2:** BvfA sequence information

## Data Availability

The original contributions presented in the study are included in the article/Supporting Material. Further inquiries can be directed to the corresponding author.
