# Supplementary figures and images for: BvrR From Brucella abortus Induces Neuroinflammation Through IRE1‐Mediated Activation of ATF2 and NF‐κB
Source: Microbiologyopen. 2026 Jan 21;15(1):e70219. doi: 10.1002/mbo3.70219 (PMC12823783; doi:10.1002/mbo3.70219)

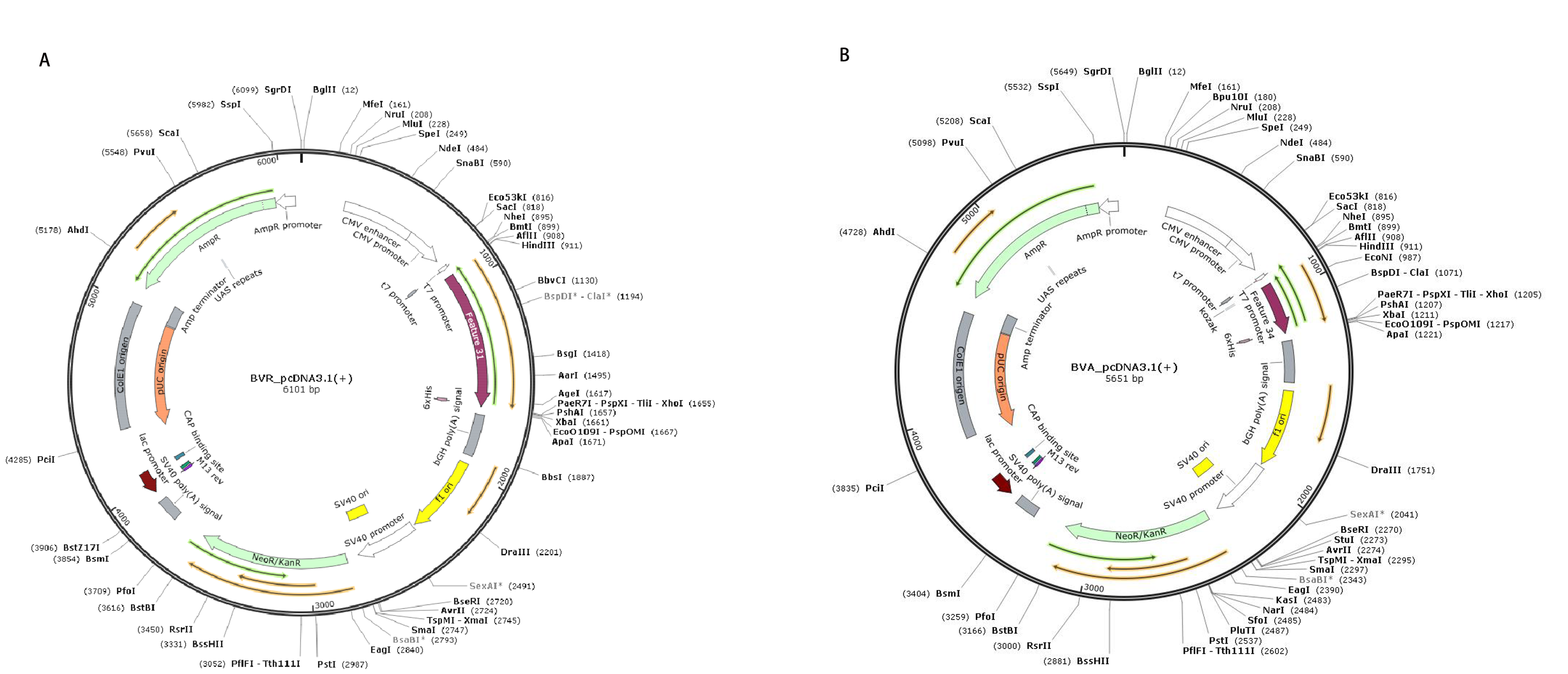

Supplement: Supplementary file 1 — Figure S1: Vector maps. [file MBO3-15-e70219-s005.tif]

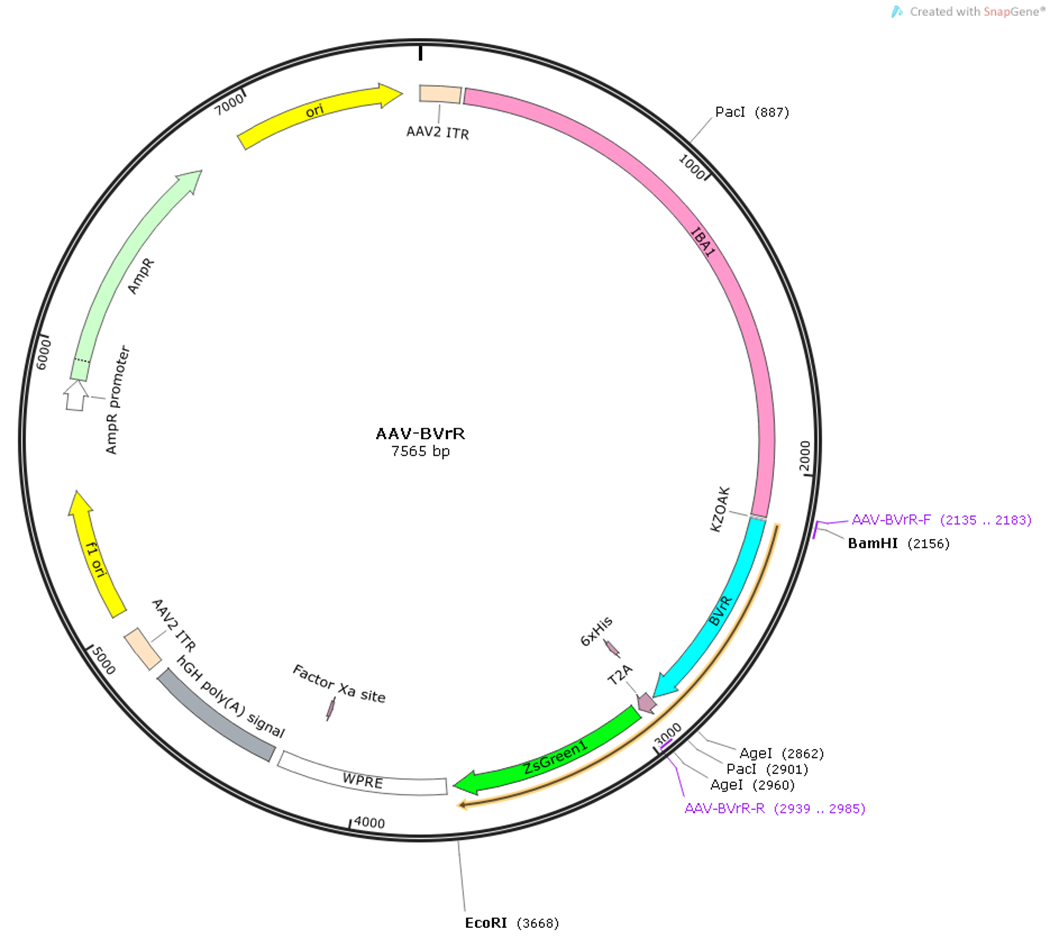

Supplement: Supplementary file 2 — Figure S2: AAV‐BvrR Vector map. [file MBO3-15-e70219-s004.tif]

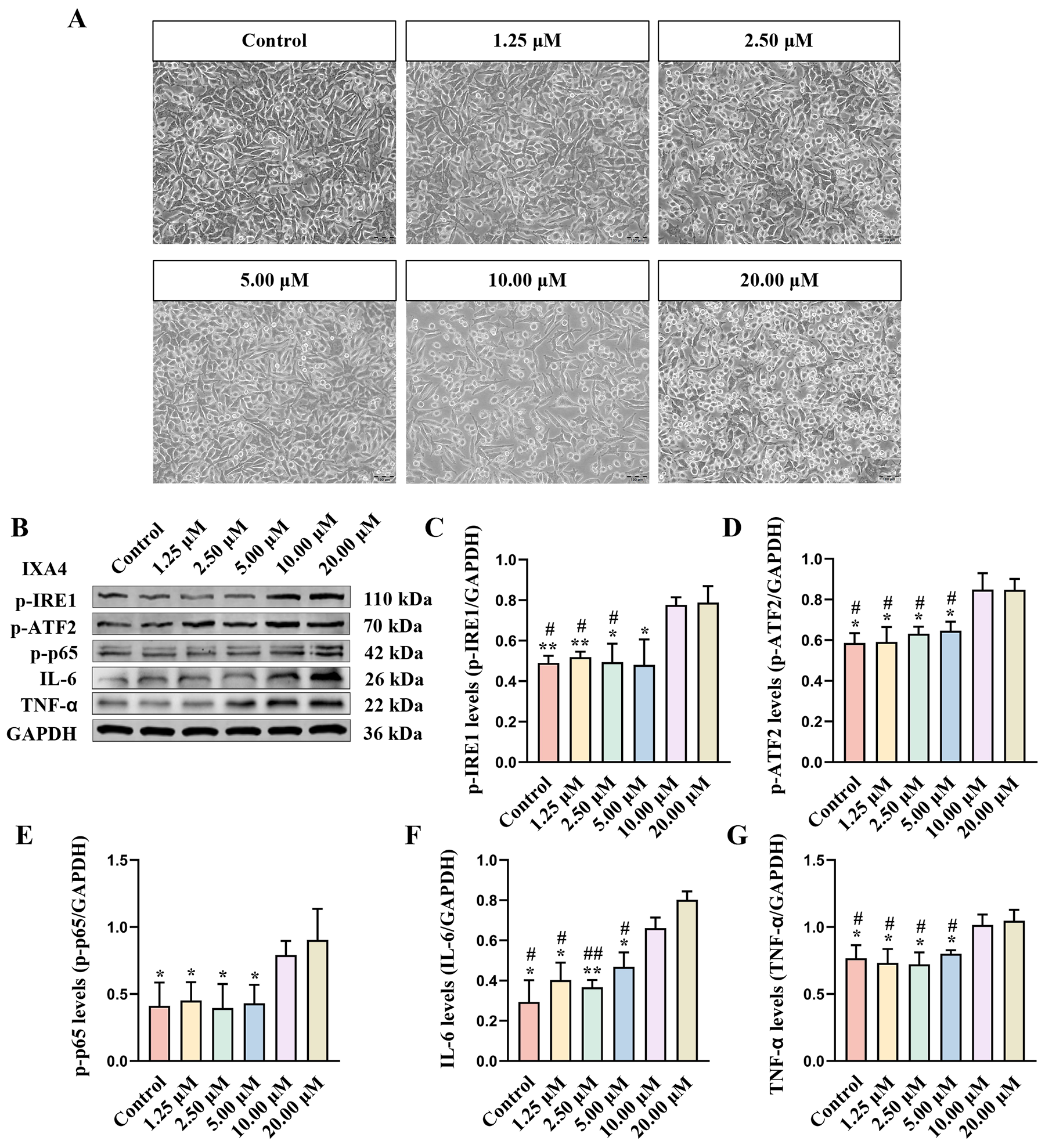

Supplement: Supplementary file 3 — Figure S3: The role of IXA4 in activating IRE1 and triggering inflammatory pathways in HMC3 cells. [file MBO3-15-e70219-s002.tif]

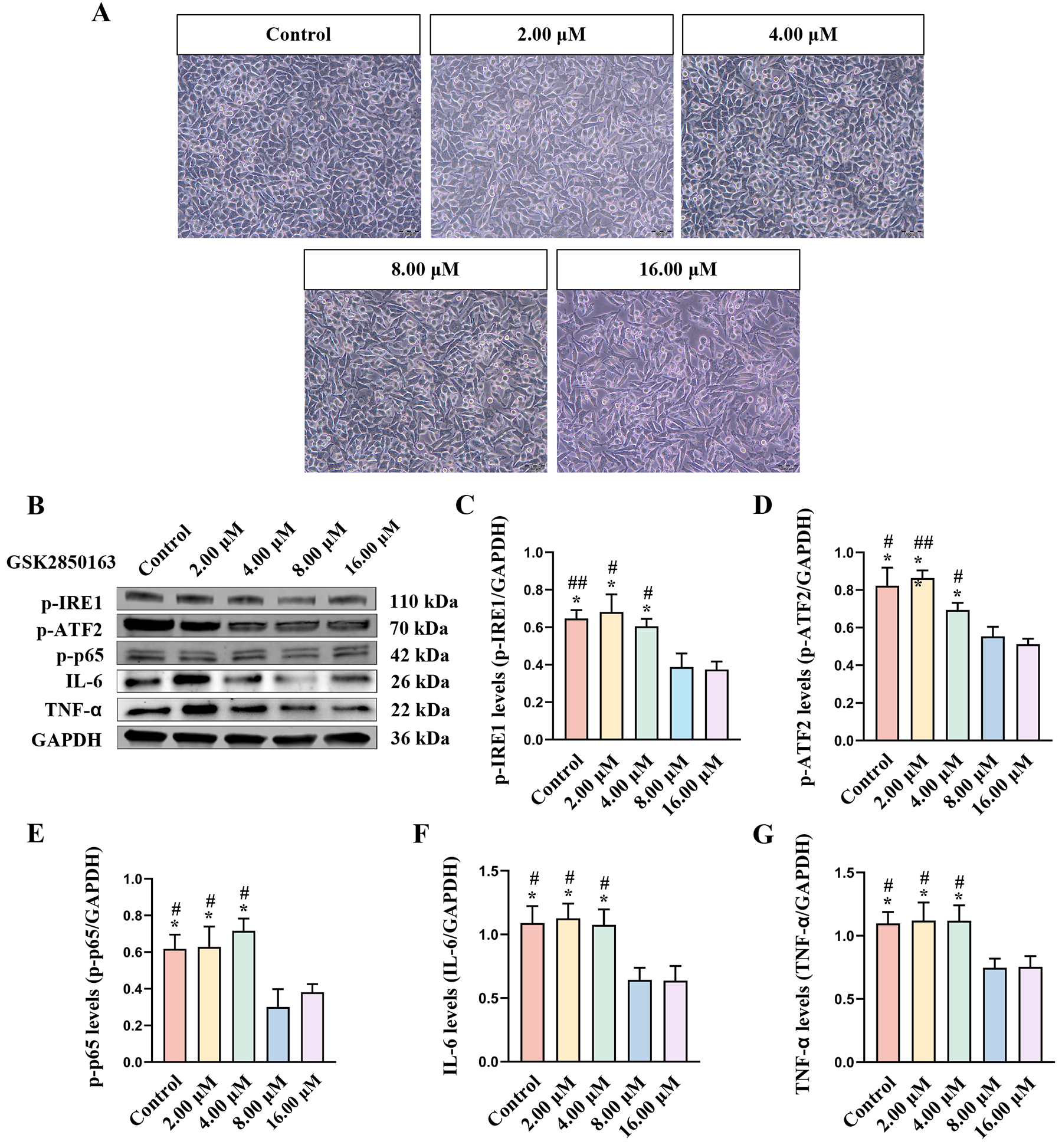

Supplement: Supplementary file 4 — Figure S4: Inhibition of IRE1, ATF2, and NF‐κB p65 activation by GSK2850163. [file MBO3-15-e70219-s003.tif]
