# Supplementary material for: BvrR From Brucella abortus Induces Neuroinflammation Through IRE1‐Mediated Activation of ATF2 and NF‐κB
Source: Microbiologyopen. 2026 Jan 21;15(1):e70219. doi: 10.1002/mbo3.70219 (PMC12823783; doi:10.1002/mbo3.70219)
Supplement: Supplementary file 5 — Table S1: BvrR sequence information. Table S2: BvfA sequence information [file MBO3-15-e70219-s001.doc]

| BvrR sequence information | ATGAAAGAGGCCTCTGCCACCCAGACCATTGCCCTGGTCGACGATGACAGAAACATCCTGACCAGCGTGAGCATCGCCCTCGAATCTGAAGGCTACAGAGTGGAAACCTACACAGACGGAGCTTCCGCCCTGGACGGCCTGATGGCCCGGCCTCCAAACCTGGCAATCTTCGACATCAAGATGCCTAGAATGGACGGCATGGAACTGCTGAGGCGGCTGAGACAGAAAAGCGACCTGCCTGTCATCTTCCTGACATCCAAGGACGACGAGATCGATGAGCTGTTTGGACTGAAGATGGGCGCCGATGATTTCATCACAAAGCCTTTCAGCCAGAGACTGCTGGTGGAAAGAGTGAAGGCCGTGCTGAGAAGAGTGGCCAGACGGCACGCCAAGCCCGCTGGCCAGCAGGCCAAGTCTCTGGAGAGAGGCCAACTGGTGATGGATCAGGAGCGGCACACATGCACCTGGAAGGGCGAGCCTGTGACCCTGACCGTGACCGAGTTCCTGATCCTGCACAGCCTGGCCCAGCGCCCCGGCGTGGTGAAAAGCAGAGATGCTCTGATGGACGCCGCTTACGACGAGCAGGTGTACGTGGACGACAGAACCATCGACAGCCACATCAAGCGGCTGCGGAAGAAGTTCAAGGCCGTTGACGATAGCTTCGAGATGATCGAGACACTTTATGGAGTGGGCTACCGGTTTAGAGAAGCCCACCACCATCACCACCACTGA |
| --- | --- |

**Table S1 BvrR sequence information**

| BvfA sequence information | ATGGCCGAGGCCCAGGTGCGGAAAGATGTGTCCGAATACGCCGGAAGACGGTGCAACACCCCTCCTAGAGGCTCTGGCATCATCGTGGGCCAGTTCAGCGGCGTGGACGACAGCCCCTTCATCAGCGACGGCGACGCCCTGGTCGCCATCGATAGATACAGATGTTTTACCACAATGAGCGAGTGCAAGGGCTGGCTGTATACCATGCAGAGCAAGTACACCAACGCCGGAGCTGCTACACTGGCCAGATGCATCAAGCGGCACCATCACCACCACCACTGA |
| --- | --- |

**Table S2 BvfA sequence information**


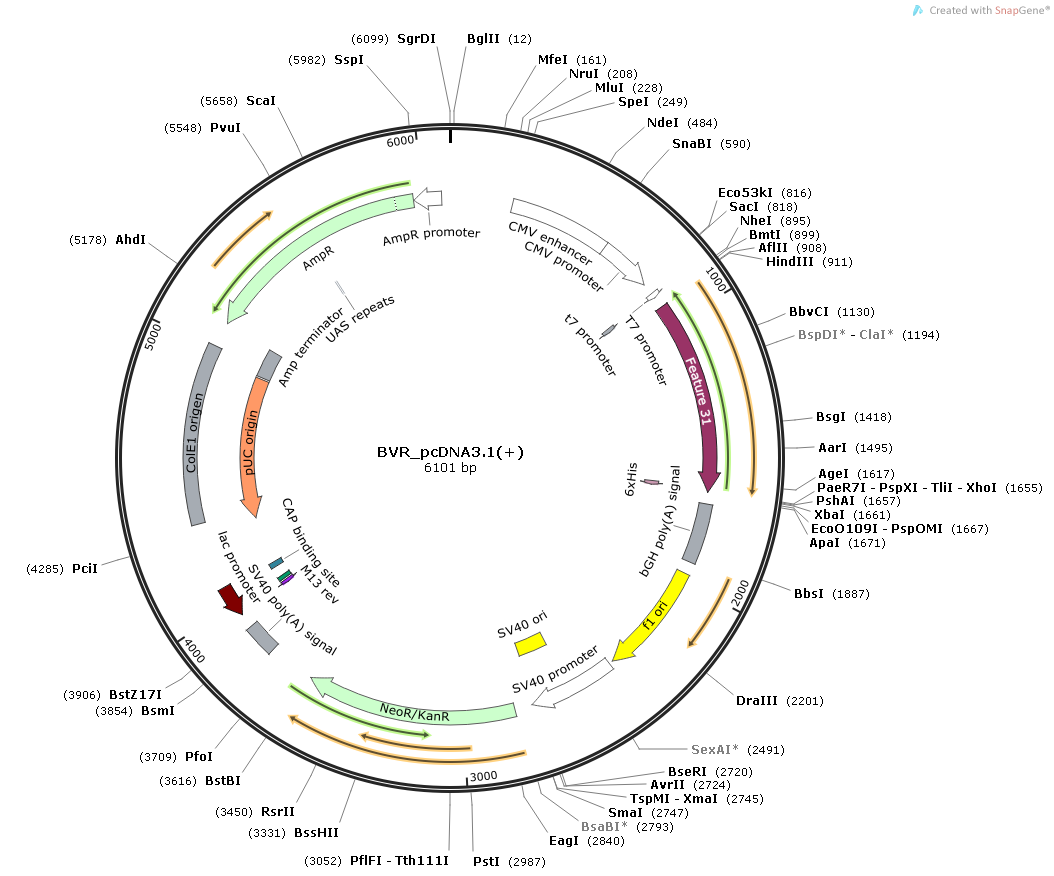

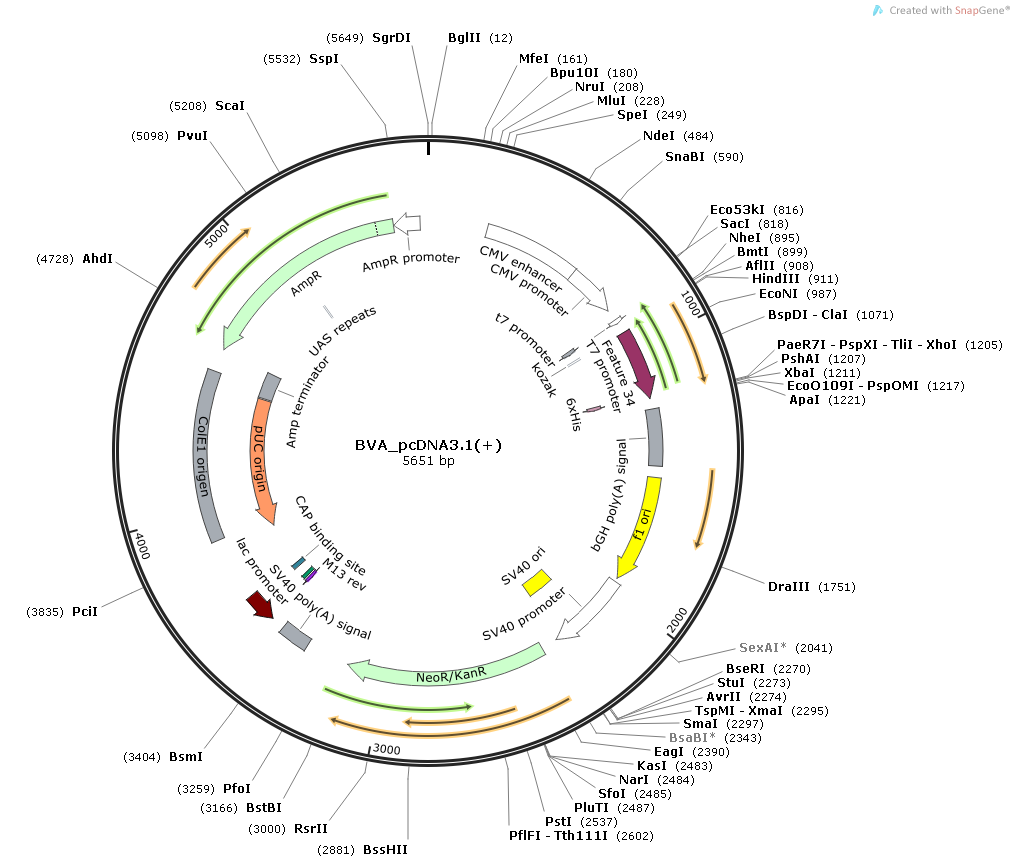


**A**

**B**

**Figure S1 Vector maps.** (A) BvrR Vector map. (B) BvfA Vector map.


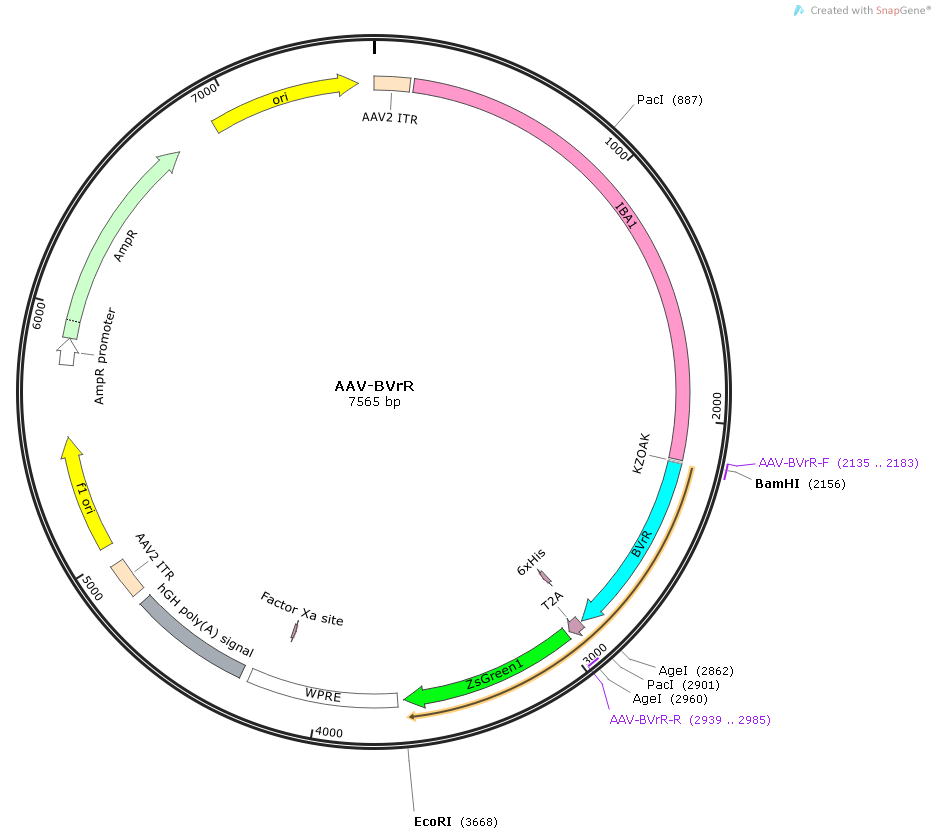


**Figure S2** AAV-BvrR Vector map.

**Results**

**S1. The effects of IXA4 on the activation of IRE1 and inflammatory pathways**

To investigate the effects of IRE1 activation on the activation of ATF2 and p65 proteins, HMC3 cells were treated with various concentrations of IXA4, a known IRE1 activator [1, 2], for 24 hours. Changes in cellular morphology were directly observed using an optical microscope. The results indicated that the HMC3 cells in the 10.0 μM and 20.0 μM IXA4 groups displayed increased spherical morphology, while no significant morphological differences were observed in the other treatment groups (**Figure S3A**). This suggests that high concentrations of IXA4 are likely to induce apoptosis in HMC3 cells. Subsequently, the levels of p-IRE1, p-ATF2, p-p65, IL-6, and TNF-α proteins were measured by Western blot (**Figure S3B**). The data showed a significant increase in the levels of all five proteins in the 10 μM group compared to the other groups (**Figure S3C-G**). Except for p-p65, protein levels in the 20 μM group were higher than those in the other groups, with no significant differences between the 10 μM and 20 μM groups (**Figure S3C-G**). In summary, 10 μM IXA4 effectively activates IRE1, ATF2 and NF-κB p65 signaling pathways in HMC3 cells.

**
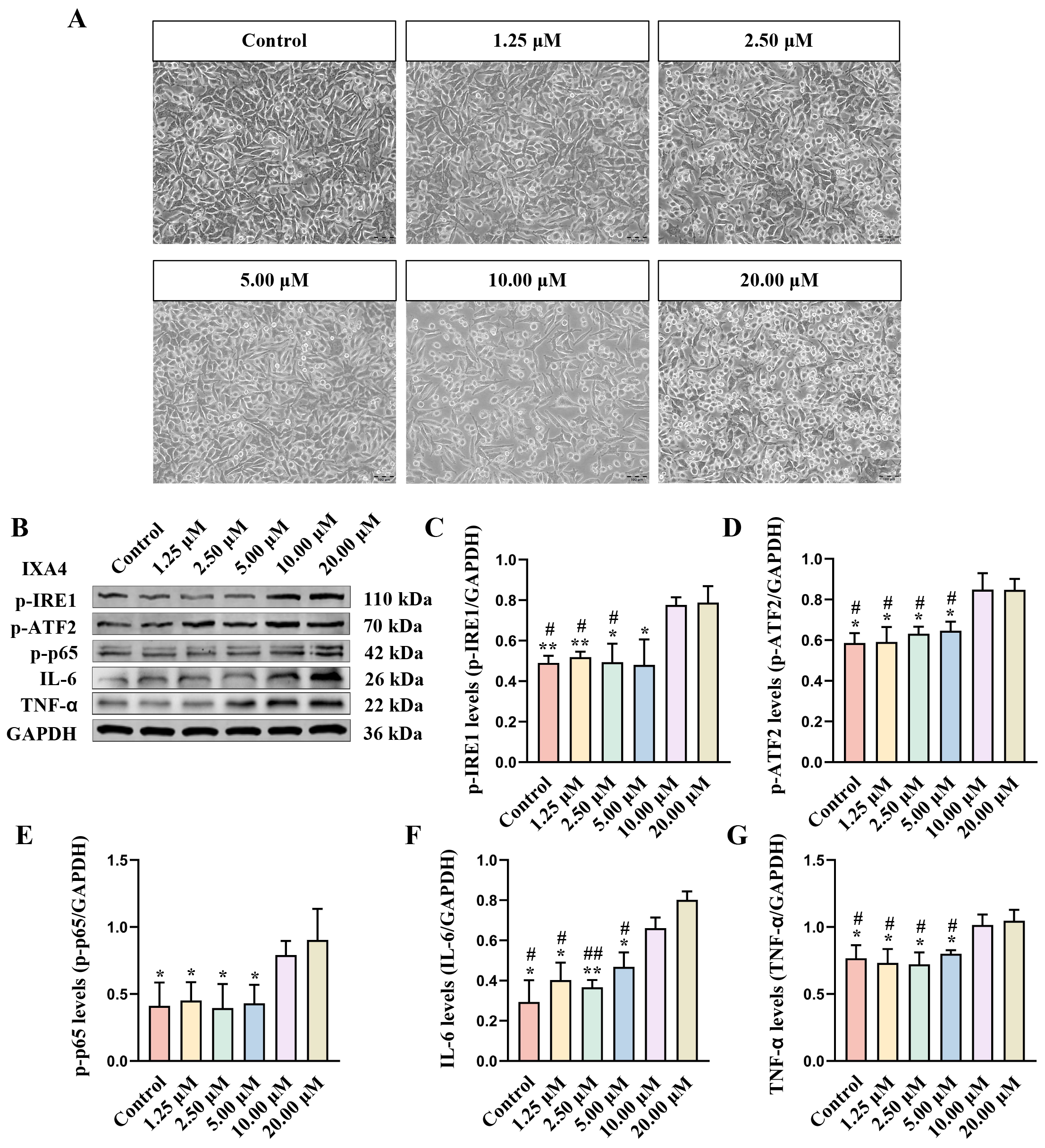
**

**Figure S3.** The role of IXA4 in activating IRE1 and triggering inflammatory pathways in HMC3 cells. (**A**) Morphological observations of HMC3 cells in each concentration group under light microscopy. (**B**) Western blot analysis of p-IRE1, p-ATF2, p-p65, IL-6, and TNF-α protein levels in different treatment groups. (**C-G**) Quantitative analysis of the relative protein levels of p-IRE1, p-ATF2, p-p65, IL-6, and TNF-α, with GAPDH as an internal standard. All experiments were repeated three times biologically, and the results are expressed as means ± standard deviations; ^*^*P* < 0.05, ^**^*P* < 0.01 versus 10 μM; ^#^*P* < 0.05, ^##^*P* < 0.01 versus 20 μM.

**S2. The effects of GSK2850163 on the inhibition of IRE1 and inflammatory pathways**

To examine the effects of IRE1 inhibition on the ATF2/IL-6 and NF-κB p65/TNF-α pathways, HMC3 cells were treated with various concentrations of GSK2850163, an IRE1 kinase inhibitor, for 24 hours [3, 4]. Changes in cell morphology following GSK2850163 treatment were observed under an inverted optical microscope, with no noticeable variation in morphology across the treatment groups (**Figure S4A**). Subsequently, levels of p-IRE1, p-ATF2, p-p65, IL-6, and TNF-α proteins were analyzed by Western blot (**Figure S4B**). The results indicated that both the 8 μM and 16 μM treatment groups exhibited significantly lower levels of these proteins compared to the three lower concentration groups (**Figure S4C-G**). However, no significant differences in protein levels were observed between the 8 μM and 16 μM groups (**Figure S4C-G**). These results suggest that 8 μM GSK2850163 effectively inhibits the activation of IRE1, ATF2/IL-6, and NF-κB p65/TNF-α pathways in HMC3 cells.


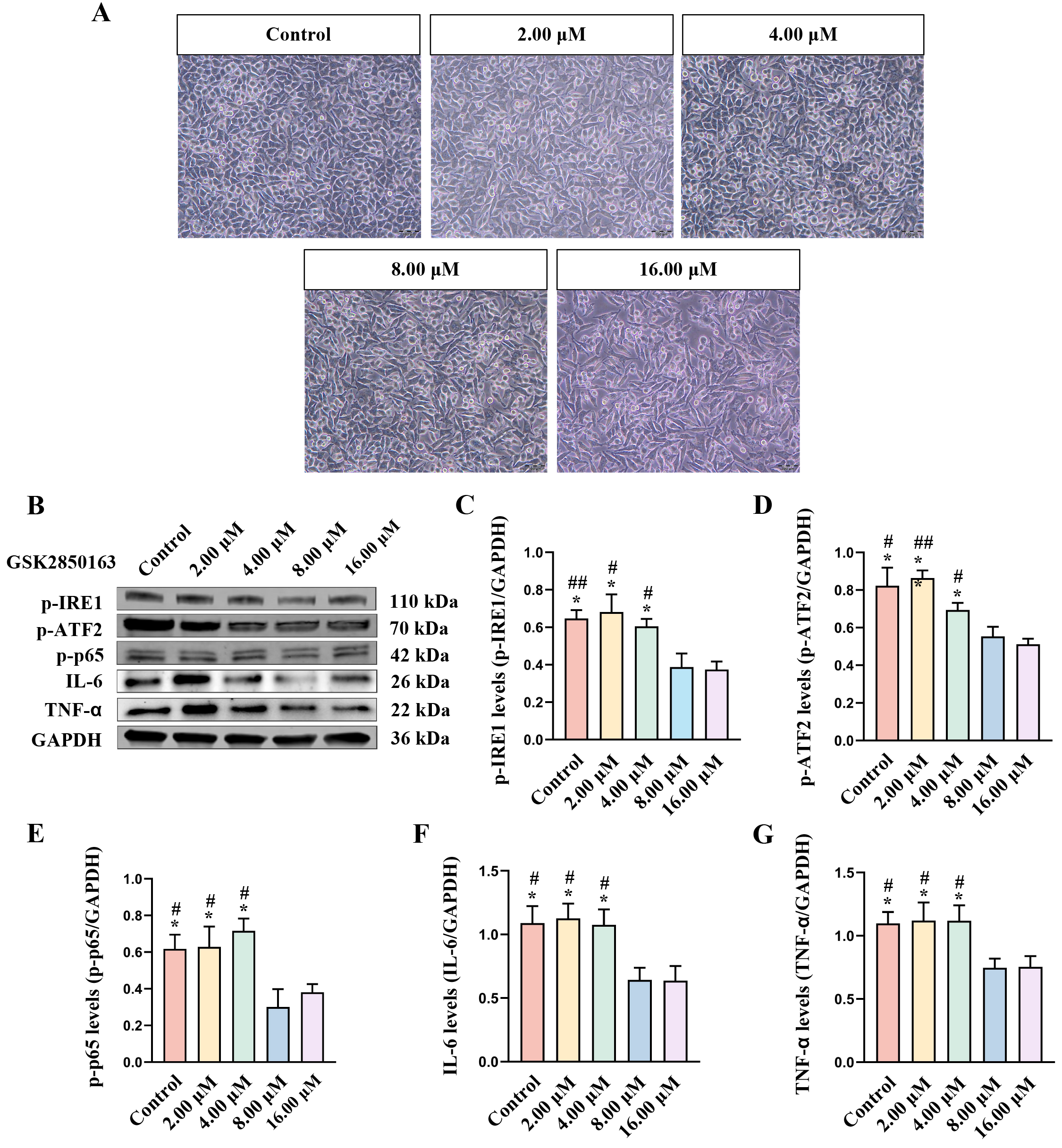


**Figure S4.** Inhibition of IRE1, ATF2, and NF-κB p65 activation by GSK2850163. (**A**) Morphological observations of HMC3 cells in each concentration group under light microscopy. (**B**) Western blot analysis of p-IRE1, p-ATF2, p-p65, IL-6, and TNF-α protein levels in different concentrations of GSK2850163 treatment groups. (**C-G**) Quantitative assessment of the relative levels of p-IRE1, p-ATF2, p-p65, IL-6, and TNF-α proteins normalized to GAPDH from Western blot analysis. All experiments were repeated at least three times, and data are presented as means ± standard deviation; ^*^*P* < 0.05, ^**^*P* < 0.01 versus 8 μM; ^#^*P* < 0.05, ^##^*P* < 0.01 versus 16 μM.

**References**

1. Fernández, J.J.; Mancebo, C.; Garcinuño, S.; March, G.; Alvarez, Y.; Alonso, S.; Inglada, L.; Blanco, J.; Orduña, A.; Montero, O.; et al. Innate IRE1α-XBP1 activation by viral single-stranded RNA and its influence on lung cytokine production during SARS-CoV-2 pneumonia. Genes Immun. 2024, 25, 43-54. doi: 10.1038/s41435-023-00243-6.
2. Fu, B.; Xiong, Y.; Sha, Z.; Xue, W.; Xu, B.; Tan, S.; Guo, D.; Lin, F.; Wang, L.; Ji, J.; et al. SEPTIN2 suppresses an IFN-γ-independent, proinflammatory macrophage activation pathway. Nat. Commun. 2023, 14, 7441. doi: 10.1038/s41467-023-43283-2.
3. Wang, X.C.; Zhou, Y.; Chen, H.X.; Hou, H.T.; He, G.W.; Yang, Q. ER stress modulates Kv1.5 channels via PERK branch in HL-1 atrial myocytes: Relevance to atrial arrhythmogenesis and the effect of tetramethylpyrazine. Heliyon. 2024, 10, e37767. doi: 10.1016/j.heliyon.2024.e37767.
4. Tian, M.; Yin, Y.; Lian, Z.; Li, Z.; Song, M.; Hu, H.; Guan, X.; Ding, C.; Wang, S.; Li, T.; et al. A rough Brucella mutant induced macrophage death depends on secretion activity of T4SS, but not on cellular Txnip- and Caspase-2-mediated signaling pathway. Vet. Microbiol. 2020, 244, 108648. doi: 10.1016/j.vetmic.2020.108648.
